# Design and test of controlling frequency circulating fan system in bulk curing barn

**DOI:** 10.1016/j.heliyon.2024.e34688

**Published:** 2024-07-20

**Authors:** Jianan Wang, Guangting Yin, Xiaolong Chen, Hongtao Shen, Weidong Duan

**Affiliations:** a College of Tobacco Science, Henan Agricultural University, Zhengzhou, 450002, China; b China Tobacco Henan Industrial CO., LTD., Zhengzhou, 450000, China

**Keywords:** Bulk curing barn, Circulating fan, Variable-frequency drive, Wind speed

## Abstract

**Objective:**

Scientific control of wind speed between tobacco leaves can improve quality of tobacco after cured in bulk curing barn.

**Method:**

Regarding the light orange color after cured tobacco and high power consumption caused by inaccurate control of the current circulating fan, and different requirements for wind speed during the tobacco curing, this paper designed a variable frequency drive system (VDS) to control the rotation speed of the circulating fan. With the design of anti-interference components, the VDS is integrated with the curing controller.

**Results:**

The curing adoption of anti-interference for the VDS device will not affect the normal operation of the tobacco curing controller during tobacco curing. Compared with the traditional circulating fan with high speed and low speed options, the VDS device can accurately control the air speed during the tobacco curing process, and improve the economic characters and internal quality of cured tobacco. This paper is also to provide an alternative method in terms of ventilation and humidity removal for industrial and agricultural production.

## 1 Introduction

The circulating fan is the heart of bulk curing barn (BCB) for flue-cured tobacco (*Nicotiana tabacum* L.) curing. It is used for exchanging heat and remove the humidity. However, the traditional control on wind speed of circulating fan with only high and low gears during tobacco curing process are not matched perfectly, resulting in undesired yellow color, unstable temperature and humidity, and loss of tobacco aroma substance [1]. The negative influence on the quality of flue-cured tobacco by wind speed has been reported extensively worldwide [2,3]. In response to this situation, Liu Guanghui [4] and Liu Chuang et al. [5] adopted two set variable frequency technology to control the wind speed during the yellowing and leaf drying of tobacco, and found that the quality of flue-cured tobacco was improved. Subsequently, He Yahao et al. [6] used the same method to keep the circulating fan at 38 °C and 54 °C, which can affect the chemical composition of the cured tobacco leaves. To further explore the influence of different speeds on the later stage of curing, Xu Qi et al. [7] and Zhou Xiaofeng et al. [8] analyzed the relationship between the rotation speed of the circulating fan and the aroma of cured tobacco leaves and found that the variable frequency circulating fan can increase the content of neutral aroma substances in cured tobacco leaves.

**Fig. 1:**
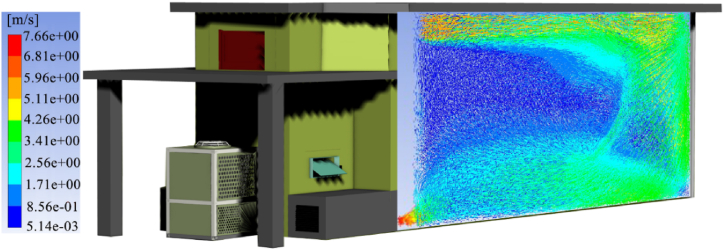
Flow vector diagram of wind speed of current BCB in leaf drying stage [13]

**Fig. 2:**
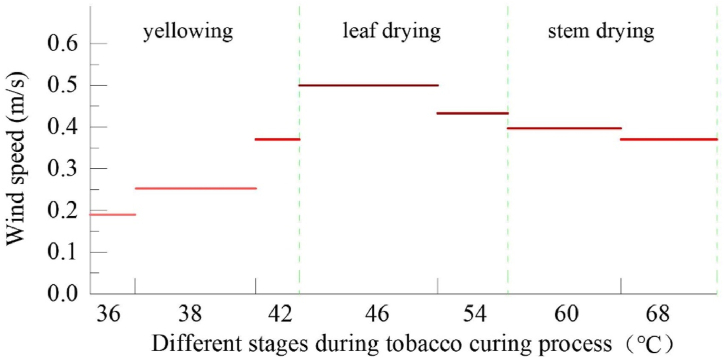
Demand for wind speed between leaves during tobacco bulk curing of different stages [14].

In China, BCBs constructed in compliance with the No. 418 standard issued by Chinese State Tobacco Monopoly Administration are widely adopted for the curing of flue-cured tobacco [9]. The overall size of the barn (Fig. 1) is 11440 × 2700 × 3500 mm (length × width × height), whose construction quantity is approximately one million units at present [[10], [11], [12]]. The demand for wind speed between leaves at different stages during the curing process of tobacco leaves is shown in Fig. 2. The wind speed in the tobacco-loading chamber increases from 0.18 m/s to 0.55 m/s and then decreases. In the current curing process of tobacco, a No. 7 axial flow fan with a speed of 1440 r/min (high gear) and 960 r/min (low gear) is commonly used to control the ventilation and dehumidification in the BCB. Fig. 1 shows the wind speed and flow vector diagram of the circulating fan currently used during the leaf drying stage, which is much higher than the required wind speed for tobacco leaf curing. It also shows an unbalanced distribution among the leaves in the tobacco-loading chamber. Therefore, there is a large difference between the wind speed required in real terms and the theoretical parameter, which may result in bad tobacco, stiff leaf, and tobacco without sufficient aroma.

At present, there are many types of frequency converters in China, and the control methods mainly include vector control frequency conversion [15], variable voltage and variable frequency (VVVF) conversion [16], and direct torque control frequency conversion [17]. There are inherent shortcomings such as low input capacity, high harmonic current and inappropriate DC circuit capacitance [18]. When the variable frequency value obtained in other regions is adopted on flue-cured tobacco curing controller (FTCc) without a suitable variable frequency drive, the rotation speed of the circulating fan may differ under the same frequency, resulting in large deviations in the result of tobacco leaf curing, and after variable frequency circulating fans are extensively adopted in the loading chamber of BCB, it was found that variable frequency drive usually generates interference to the stability of data collection and transfer of FTCc [19]. The variable frequency control of circulating fan in BCB becomes an intrinsic requirement for fine production of tobacco in the modern agriculture [20]. However, there are only few studies on the control principle and process of frequency conversion technology. Based on the control theory of blade tip wind speed and the requirement for ventilation and humidity removal of flue-cured tobacco, this paper initiated the research and development of variable frequency drive system (VDS) to control circulating fan for the fine production of flue-cured tobacco in the new era.

## 2 System design and control

### 2.1 Type and control of variable frequency drive

At present, BCBs in China generally use 50 Hz grid power supply. The circulating fan uses 2.2 kW, the total pressure is 170–250 Pa, and the air volume is above 15,000 m^3^/h [9]. For different curing stages, this study used VDS to reduce the output power and rotation speed of circulating fan. According to the production specification of tobacco leaf curing, the output wind speed can be varied from 0–15,000 m^3^/h by adjusting the frequency converter to change from 0 Hz to 50 Hz to adjust the wind speed.

### 2.2 Selection of variable frequency drive

Through the anti-electromagnetic interference test in the laboratory, the model 9600 VDS manufactured in Fuan Weiken Motor Co., Ltd. Was selected. The VDS adopts open-loop vector control with a range from 0 Hz to 650 Hz V/F, the frequency command resolution of 0.1 Hz, and the acceleration time and deceleration time of 1 s. When the grid voltage changes, it can automatically keep a stable and constant output voltage.

### 2.3 Mechanism of flue-cured tobacco frequency conversion regulation

At present, the FTCc is widely used to control the entire process of tobacco curing. Therefore, the variable frequency control needs be re-developed with single-chip microcomputer (SCM) to establish a theoretical wind speed data model and to add signal input and output terminals. As the data model showed in Fig. 2, the dry-bulb temperature (DBT) set for the different curing stages is taken as differentiating value**,** the database for wind speed control is established in the SCM, and the current wind speed is monitored by the wind speed collection device. The SCM analyzes the actual wind speed between leaves in the loading chamber based on the collected data and the standard wind speed value. The SCM controls the U, V and W current terminals at the output end of the VDS through R, S and T to adjust the rotation speed of circulating fan and control the ventilation of the curing chamber.

**Fig. 3:**
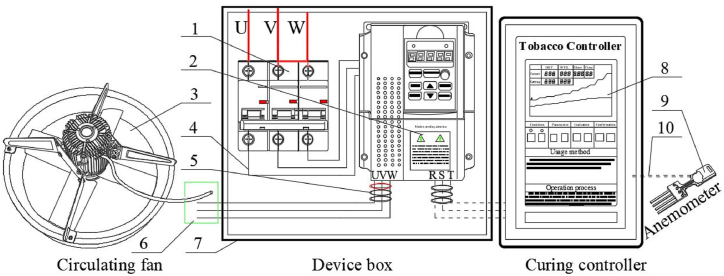
Composition and line connection of variable frequency control device 1. Power air switch, 2. Inverter, 3. Circulating fan, 4. Power cord, 5. Anti-interference coil, 6. Junction box, 7. Anti-interference box, 8. FTCc, 9. Anemometer, 10. Signal line.

Fig. 3 shows the composition and line connection of variable frequency control device. The power line of circulating fan is connected to the VDS through an anti-interference box. The VDS is installed with a power input, a signal input, and a power output. The anemometer is connected to the FTCc from the input port. The anemometer is installed in a representative position between the leaves in the loading chamber to detect the real-time wind speed between the tobacco leaves during the curing process. To improve the anti-interference of the VDS, all connected signal and power lines adopt anti-interference equipment, such as anti-interference rings, boxes or twisted wires with shielding sleeves, etc.

### 2.4 Control process and logic of variable frequency drive

**Fig. 4:**
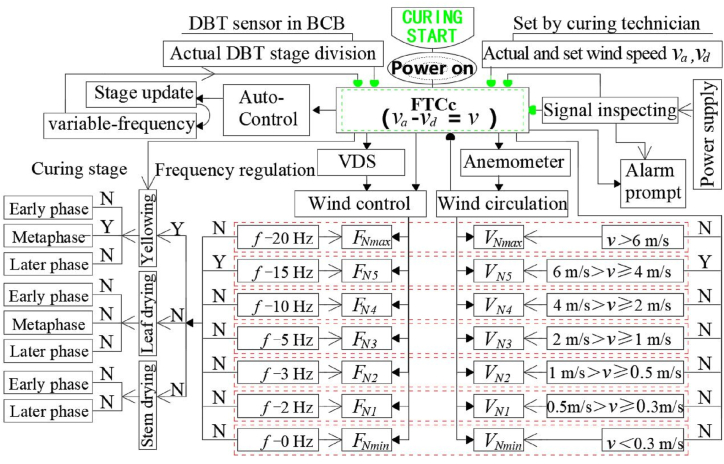
Control flow of VDS technology for tobacco curing.

The FTCc is controlled by traditional SCM of STM32F103RBT6. The VDS wind control has two modes, including automatic control in expert mode, and manual control by curing technicians. The logic of VDS control in the curing process is shown in Fig. 4. The expert mode is a pre-set program based on the position of the tobacco leaves, loading capacity, the humidity content of the tobacco leaves. And then the frequency of each curing stage is set according to the program. After the curing starts under manual mode, the FTCc recognizes the temperature in the curing room through the installed DBT and humidity sensors, and uses the DBT as a reference to divide different curing stages, including yellowing stage, leaf drying stage, and stem drying stage. The actual DBT *v*_*a*_ of the anemometer installed in the tobacco chamber is input to the FTCc through the data transmission line. The FTCc will automatically match the standard value of wind speed *v*_*d*_, and calculate the difference *v* between *v*_*a*_ and *v*_*d*_.

Then the FTCc outputs signal to VDS that adjusts the voltage and current of the circulating fan to control the rotation speed of the fan. The 7 *x* speed gear values are *v* < 0.3 m/s, 0.3 m/s ≤ *v* < 0.5 m/s, 0.5 m/s ≤ *v* < 1 m/s, 1 m/s ≤ *v* < 2 m/s, 2 m/s ≤ *v* < 4 m/s, 4 m/s ≤ *v* < 6 m/s and *v* > −0.16 m/s, corresponding to f - 30 Hz, f - 15 Hz, f - 10 Hz, f - 5 Hz, f - 3 Hz, f - 2 Hz, and f - 0 Hz, respectively. For the original input power of 50 Hz, the FTCc will automatically subtract the output frequency of 0–30 Hz from the highest frequency. The greater the wind speed between the leaves than the required value, the greater the adjustment coefficient of the circulating fan after the gears are switched, and vice versa. When the sensors detect that the wind speed between the leaves is lower than 10 % of the required value, the FTCc will automatically adjust frequency of the output circulating fan to 50 Hz.

The anemometer is not needed under the expert control, as known as programmed control mode, which is applicable to the year-round tobacco curing process. But the anemometer is required for manual mode, as known as the variable frequency development mode matched with specific curing process, which is applicable to the tobacco leaves that requires specific curing environment.

## 3 Equipment installation and testing

### 3.1 Test material

From 2021 to 2022, the tests were carried out at the Tuanjie Tobacco Station in Cangyuan County and Dedang Tobacco Station in Yongde County of Lincang City, and Songfeng Tobacco Station in Xiangcheng County of Xuchang City.

The tobacco varieties named Zhongyan 100 was selected and the matured middle leaves were taken as the test samples that have consistent size, shape, weight, initial water content, growth period, and yellowing. The tests conforms to the local custom in terms of tobacco sticks and loading density in the loading chamber of BCBs. The BCB is constructed in compliance with the requirement for airflow descending barn specified in the No. 418 document of Chinese State Tobacco Monopoly Administration [9]. The tests used an air-source heat pump for heating, and the curing process was implemented in accordance with local technical standards. The tests were to compare the VDS circulating fan with the control treatment (CK) of circulating fan as the control treatment with the traditional control on wind speed of circulating fan using only high and low gears largely adopted in local BCBs. The VDS circulating fan was installed, debugged and tested by Xuchang Tongxing Modern Agricultural Technology Co., Ltd. And each treatment was set 3 repetitions in each location.

### 3.2 Detection items

The experimental data were recorded and calculated including loading fresh tobacco leaves volume, total volume of cured tobacco leaves, coal consumption, electricity consumption, coal consumption per kilogram of cured tobacco leaves, electricity consumption per kilogram of cured tobacco leaves, coal consumption cost per kilogram of cured tobacco leaves, electricity consumption cost per kilogram of cured tobacco leaves, and the total energy consumption per kilogram of cured tobacco leaves.

During the flue-curing process, a low-frequency anti-interference instrument (NSG1060, Shenzhen Haogu) was used to detect the signal interference before and after the anti-interference measures were taken for the VDS controlling the circulating fan.

At the key points of the DBT in the tobacco curing process including 38 °C, 42 °C, 47 °C, 54 °C, 60 °C and 68 °C, an anemometer (WD4150C2, Hangzhou Jili, China) was used to detect the wind speed in the tobacco loading chamber of the BCB every 6 h at each key points of the DBT. The anemometer was installed at 2.5 m from the insulation wall, in the middle of the right beams.

After the tobacco curing process was finished in the BCB, the tobacco grade characteristics of 5 sticks of tobacco leaves in the same loading location were classified by professional according to the Chinese national standard of flue-cured tobacco classification coded with GB2635-1992. Statistics of economic benefits was contrasted according to the purchase price of tobacco grade market.

### 3.3 Data processing

The GraphPad Prism 5.0 (U S. GraphPad Software Company, San Diego, California) was used for data analysis and automatic figure generation.

## 4 Results and analysis

### 4.1 Anti-interference effect of VDS

**Fig. 5:**
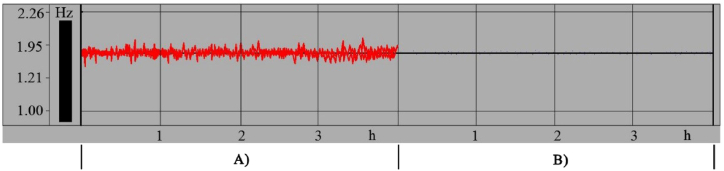
Comparison before and after implementation of anti-interference measures for variable-frequency drive A) before implementation, B) after implementation.

Fig. 5 shows the interference before and after applying anti-interference measures to the VDS to control the circulating fan. Before the implementation of the anti-interference measures (Fig. 5A), it shows a continuous sound wave interference signal, affecting the FTCc to collect and transmit the data of DBT, humidity and wind speed in the BCB. Meanwhile, the data distortion occurred time to time on the display screen. After adopting the anti-interference measures to the VDS (Fig. 5B), the discontinuous interference was distributed in the form of dots, showing that the anti-interference measures adopted in the test will not affect the normal work of the FTCc when the tobacco leaves were curing in the loading chamber of BCB.

### 4.2 Comparison of wind speed in the tobacco curing process

**Fig. 6:**
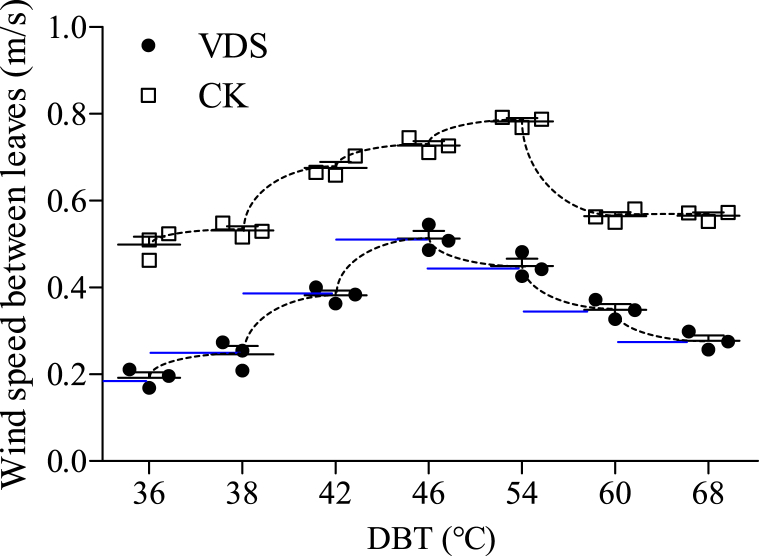
Variation of wind speed between tobacco leaves of different treatments during tobacco curing. Note: The blue line “-” represents the demand for wind speed between leaves during tobacco bulk curing of different stage whose data is originated from Fig. 2.

The change of wind speed between the leaves in the BCB is shown in Fig. 6 at the key points of the DBT in the tobacco curing process. Due to differences in leaf moisture content at different time periods, there are certain differences in wind speed between tobacco leaves at key point of the DBT. As the blue horizontal line in Fig. 6 represents the demand for wind speed between leaves during tobacco bulk curing of different stage, the wind speed between leaves is close to the theoretical value under the VDS circulating fan. However, the wind speed is obviously higher than the required standard value when using the traditional circulating fan (CK) with only high and low gears. This shows that the study plan in this paper can effectively reduce the wind speed between the leaves during the curing process and achieve the expected purpose.

### 4.3 Effects on economic characters and internal quality of cured tobacco

**Fig. 7:**
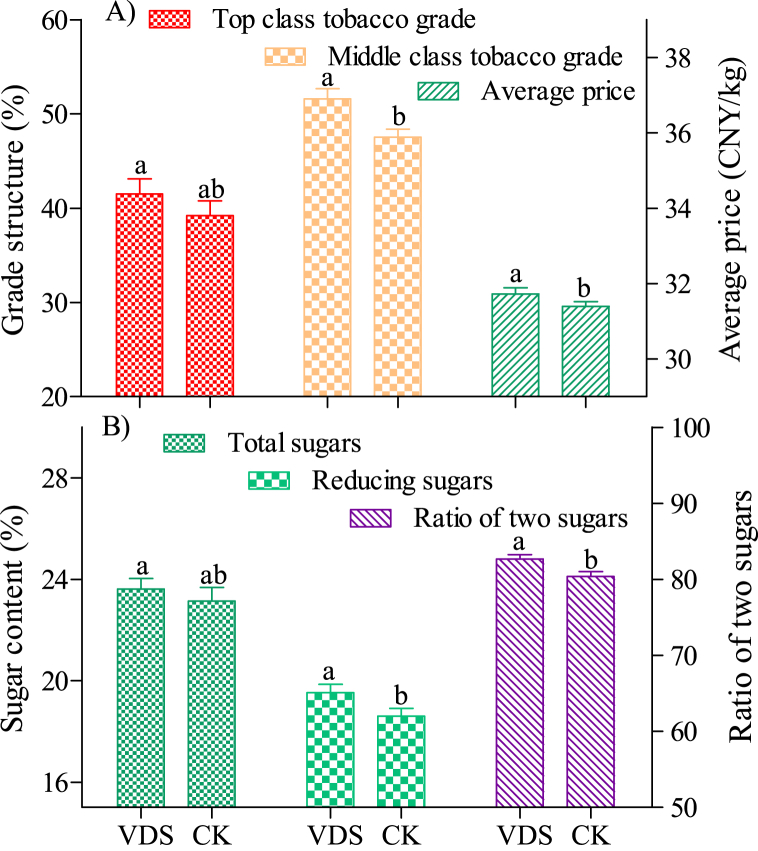
Comparison of grade structure and internal quality of cured tobacco with different treaments in Xuchang city. Notes: A) is grade structure, and B) is internal quality of cured tobacco. The letters (a, b) after the numbers in the same column indicate the test difference reached a significant level (p < 0.05), the same interpretation is used below.

Fig. 7A shows the comparison between the grade structure of cured tobacco leaves and the average selling price. The proportion of top-class tobacco grade of flue-cured tobacco (involving experimental tobacco grades of C1F, C2F、C3F, C1L, C2L), middle-class tobacco leaf (involving experimental tobacco grades of C3L, C4F, C4L, C3V and their weight) and the average selling price of VDS showed that was higher than CK, and the middle-class tobacco leaf and average selling price reached a significant difference level.

Ratio of the two sugars (reducing sugars to total sugars) is an important parameter for measuring the internal quality of flue-cured tobacco. Fig. 7B shows total sugars, reducing sugars and ratio of two sugars of the tobacco after cured. The difference of reducing sugars and ratio of two sugars between VDF and CK has reached a significant level. Generally, flue-cured tobacco has a higher ratio of two passes and a more coordinated internal quality [21], which shows VDS can improve the quality of cured tobacco.

Table 1 shows the energy consumption of tobacco leaves and the income (CNY/per batch each barn. Under the condition that the weight of fresh and dried tobacco leaf after cured was basically same, the curing time and energy consumption of VDS were higher than that of the CK, and the curing time reached a significant level. However, the average income of cured tobacco leaves per batch for each curing barn was more than 260 CNY. Based on the income of sales and energy consumption, the income per batch for each curing barn of cured tobacco leaf controlled by VDS was 207.54 CNY, which is higher than that of CK.

**Table 1:** Comparison of curing energy consumption costs under different treatments in Lincang city.

Index	VDS	CK
Total fresh tobacco (kg)	4257 ± 52.16 a	4279 ± 31.81 a
Total dry tobacco (kg)	690.59 ± 8.35 a	699.71 ± 4.99 a
Curing time (h)	151.33 ± 2.52 b	143.33 ± 1.53 a
Electricity consumption (kWh/each barn)	960 ± 21.07 a	899 ± 39.96 a
Total cost per batch for each curing barn (CNY/per batch each barn)	825.6 ± 18.12 a	773.14 ± 36.37 a
Income per batch for each curing barn (CNY/per batch each barn)	24086.52 ± 229.73 a	23826.75 ± 339.13 a

Note: In 2022, the price of the local electricity price was 0.86 CNY/kWh.

## 5 Discussions

The heat transfer and exchange in the BCB rely on the wind speed, and the uniformity of the wind speed represents the distribution of the temperature [22]. A uniform wind speed can maintain a stable temperature and humidity space for tobacco leaves and is conducive to the consistency of the tobacco curing process and the improvement of the tobacco curing quality [23]. Studies show that higher wind speeds in the barn are more likely to cause level temperature differences [24]. The outcome of this study showed significance compared with the traditional high-gear operation mode in terms of controlling wind speed in the BCB, indicating that the circulating fan with VDS will help reduce the level temperature difference between tobacco leaves in the tobacco loading chamber that is more conducive to the consistency of yellowing, dehydration, and appearance quality of the same layer of tobacco leaves [24,25]. Meanwhile, a batch of tobacco leaves in BCB using the circulating fan with VDS can increase the quality of leaf appearance.

During tobacco curing process the tobacco curing DBT in BCB slowly rises from the previous room temperature (about 35 °C) to 68 °C with different wind speed [26,27]. The traditional wind speed controlled can also affect the decomposition and synthesis of substances of tobacco leaves. To explore the influence of wind speed on the quality of tobacco, Sun et al. found that the optimal wind speed can fast the degradation of macromolecular substances and facilitate the sugar converting into more aroma substances, and significantly improve the inherent quality of tobacco leaves [28]. In general, tobacco leaves after cured with better intrinsic quality have a higher ratio of total sugars to reducing sugars [29], [30], which shows that BCB's circulating fan with VDS to cure tobacco can improve both grade quality and inherent quality. Moreover, research is needed on the effect of wind speed controlled on enzyme activity.

## 6 Conclusion

Facing the insufficient control of wind speed of circulating fan in BCB, this paper developed a device for controlling the rotation speed of the circulating fan in the loading chamber, which is mainly composed of wind speed detection, flue-cured tobacco control programming and anti-interference components. During the flue-curing process, technicians can quickly operate and program the upgraded FTCc. Compared with the traditional circulating fan with only high and low gears, it can accurately control the wind speed between the leaves at different stages during the tobacco curing process, the anti-interference measures will not affect the normal operation of the FTCc during the curing process, and improve the economic characters and internal quality of cured tobacco.

## Funding statement

Project Fund: Project is funded by Henan 10.13039/501100008862China Tobacco Industry Co., Ltd. “Research and application of key technologies to improve the adaptation rate of Lincang tobacco leaves and gold leaf high-end cigarettes (2021410000340261)", “Optimization and improvement of Henan tobacco leaf quality improvement and aroma curing (2022410000340099)”. We also thank Emate (www.emate.ac.cn) for their English language editing.

## Data availability statement

The data used to support the findings of this study are all in the manuscript.

## CRediT authorship contribution statement

**Jianan Wang:** Writing – original draft, Conceptualization. **Guangting Yin:** Formal analysis, Conceptualization. **Xiaolong Chen:** Methodology, Data curation. **Hongtao Shen:** Methodology, Data curation. **Weidong Duan:** Writing – review & editing, Methodology.

## Declaration of competing interest

The authors declare the following financial interests/personal relationships which may be considered as potential competing interests:Jianan Wang reports financial support was provided by 10.13039/501100008862China Tobacco Henan Industrial CO., LTD. If there are other authors, they declare that they have no known competing financial interests or personal relationships that could have appeared to influence the work reported in this paper.

## Figures and Tables

**Figure:**
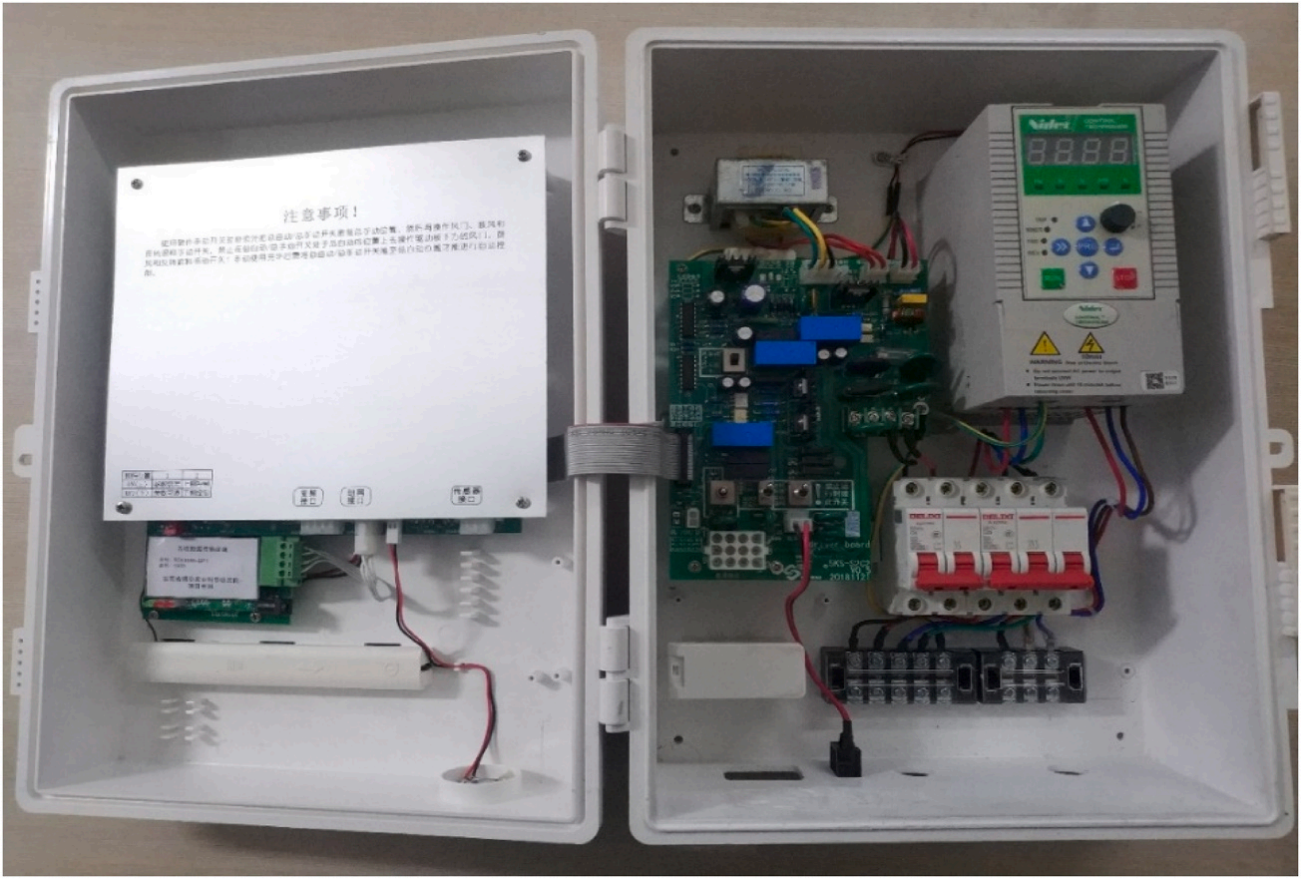


**Figure:**
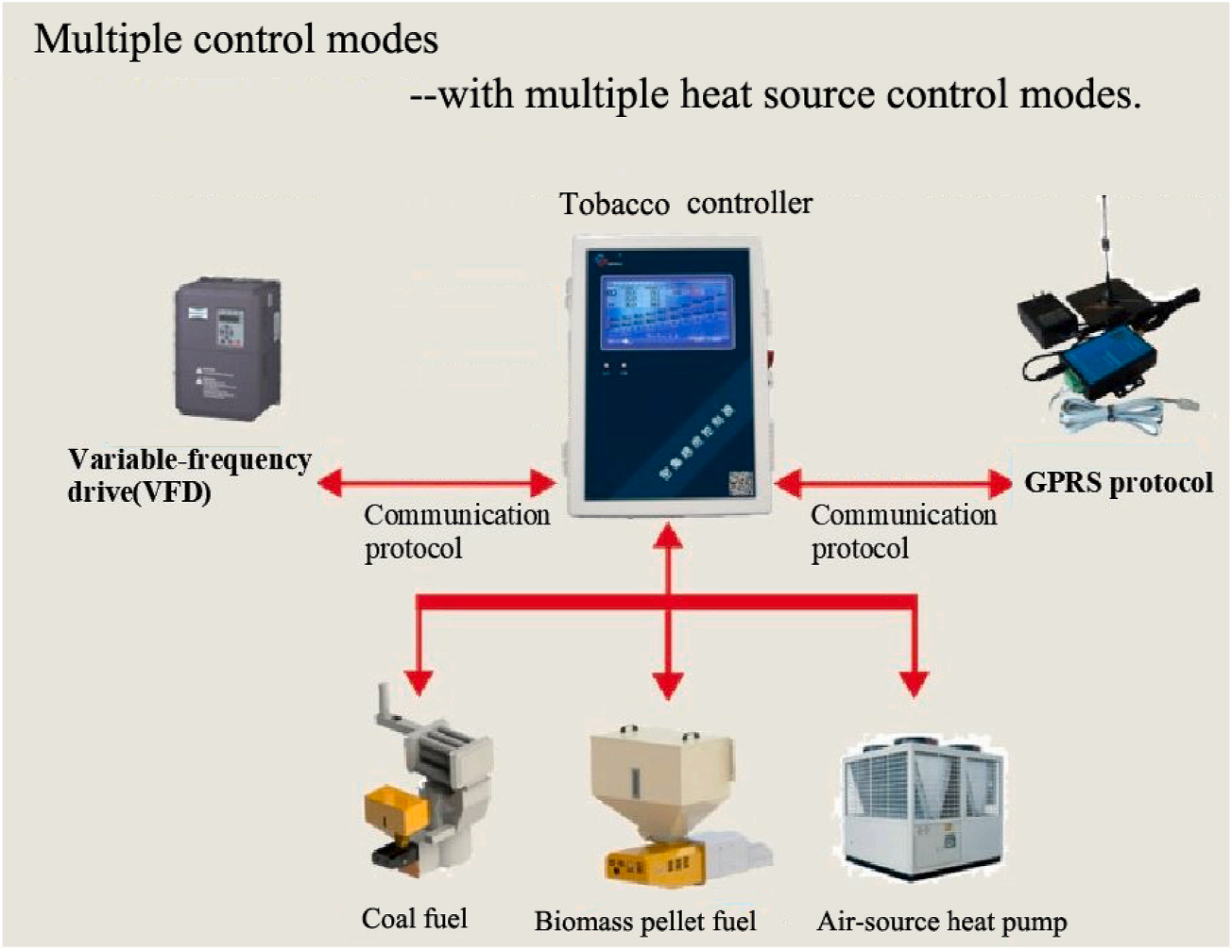


**Figure:**
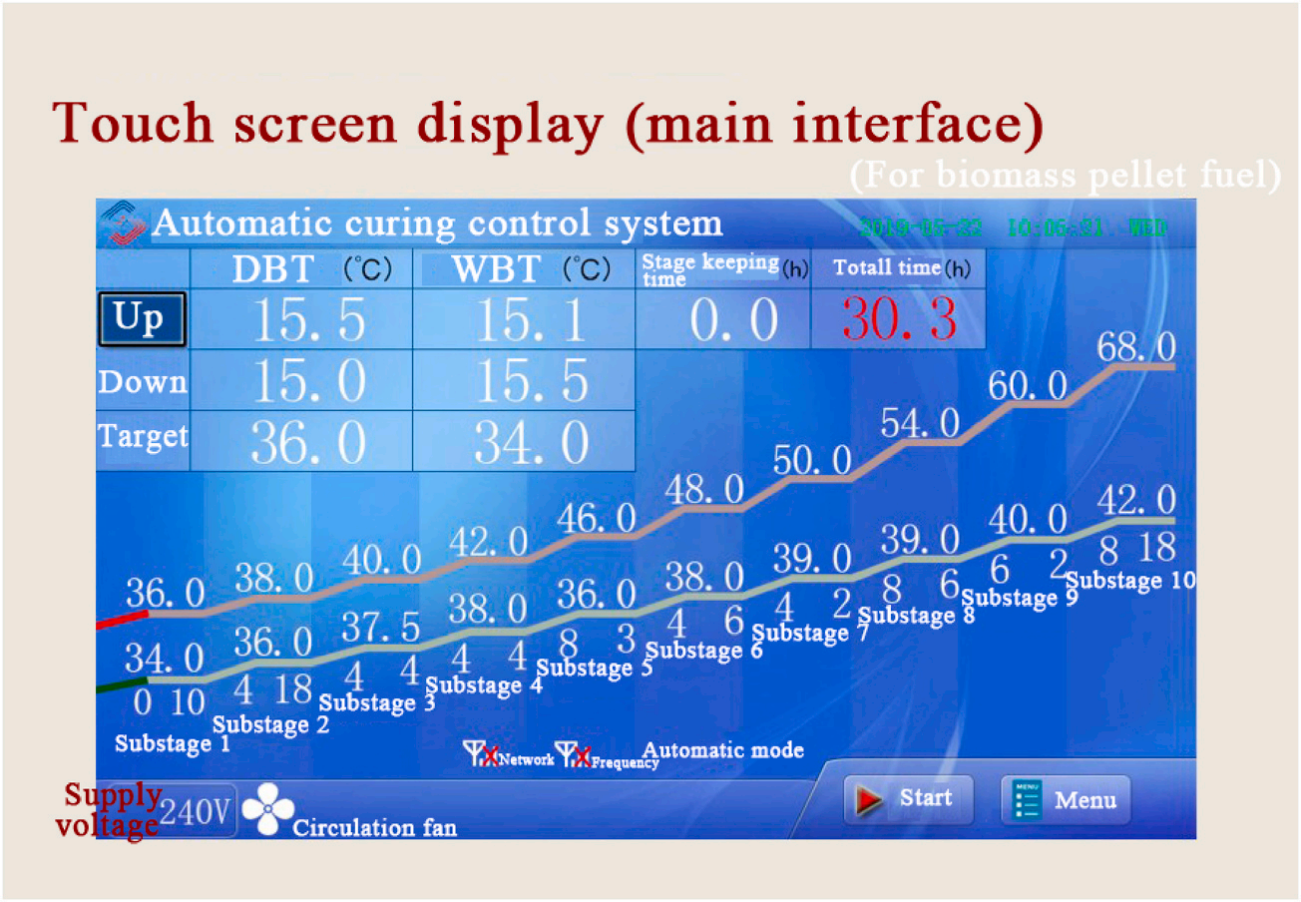


## Appendix

The frequency conversion-automatic control all-in-one machine developed.

Remote intelligent control mode with multiple heat sources.
